# An investigation of microRNA‐103 and microRNA‐107 as potential blood‐based biomarkers for disease risk and progression of Alzheimer's disease

**DOI:** 10.1002/jcla.23006

**Published:** 2019-08-16

**Authors:** Jie Wang, Chunyan Chen, Yun Zhang

**Affiliations:** ^1^ Department of Neurology Tongren Hospital, Shanghai Jiao Tong University School of Medicine Shanghai China

**Keywords:** Alzheimer**'**s disease, biomarker, disease progression, disease risk, miR‐103

## Abstract

**Background:**

This study aimed to assess the correlation of circulating microRNA‐103 (miR‐103) and microRNA‐107 (miR‐107) with disease risk and cognitive impairment of Alzheimer**'**s disease (AD).

**Methods:**

Plasma samples from 120 AD patients, 120 Parkinson**'**s disease (PD) patients (served as disease control), and 120 healthy controls were collected for miR‐103 and miR‐107 detections using real‐time quantitative polymerase chain reaction. Mini‐Mental State Examination (MMSE) score was documented and was used to accordingly assess the dementia severity.

**Results:**

miR‐103 expression was decreased in AD patients compared with PD patients and healthy controls, and receiver operating characteristic (ROC) curve analyses illustrated that it was able to differentiate AD patients from PD patients and healthy controls. Additionally, miR‐103 positively correlated with MMSE score and negatively correlated with dementia severity in AD patients. miR‐107 expression was lower in AD patients compared with healthy controls but similar between AD patients and PD patients, and ROC curve analyses revealed that it was able to differentiate AD patients from healthy controls but not AD patients from PD patients. miR‐107 was positively correlated with MMSE score and negatively correlated with dementia severity in AD patients, while the correlation coefficient of miR‐107 with MMSE score was lower than that of miR‐103 with MMSE score. Besides, miR‐103 was positively correlated with miR‐107 in AD patients, PD patients, and healthy controls.

**Conclusion:**

miR‐103 may be a better choice than miR‐107 to serve as a potential biomarker for disease risk and disease progression of AD.

## 1 INTRODUCTION

Alzheimer**'**s disease (AD) is an age‐related neurodegenerative disease that is pathologically characterized by β‐amyloid (Aβ) aggregation and hyperphosphorylation of tau protein that forms senile plates and neurofibrillary tangles, and clinically manifested as progressive memory loss and cognitive decline.^1^ It takes around a decade for the disease to be symptomatic, and once the disease takes course, patients often suffer from gradual decline in ability to function in daily life, which places great emotional and financial stress to the family.^2^ Although there have been well‐established diagnostic criteria for AD, the sensitivity and specificity of AD diagnosis are far from satisfactory. Knowing that the amyloid plaques and neurofibrillary tangles are pathogenic hallmarks of AD, several biomarkers such as Aβ and total tau detected in cerebrospinal fluid (CSF) are used to detect AD.^3^ However, the approach for the pathological examinations assessing these biomarkers is invasive. In addition, epigenetic factors such as RNA interference have been increasingly reported in AD pathogenesis, which are abundant and easily detectable in peripheral blood.^4^, ^5^ Therefore, a simple yet reliable blood test based on RNAs is necessary to help distinguish AD.

In recent years, small regulatory RNAs are identified in a variety of human tissues, and one of which is microRNAs (miRNAs). miRNAs are endogenous and non‐coding RNAs ranging from 18 to 24 nucleotides long.^6^ They bind in the imperfect complementarity to mRNAs and cause RNA degradation or translational arrest to reduce protein expressions.^7^ In the central nervous system, the regulatory roles of miRNAs are shown in neurodegeneration, and quite a number of miRNAs are abnormally expressed in neurodegenerative diseases such as AD and Parkinson**'**s disease (PD).^8^, ^9^, ^10^, ^11^, ^12^ And the neuropathological mechanisms of miRNAs in AD involve production and increased secretion of amyloid protein precursor, regulation of neuroinflammation, and neuron apoptosis, etc.^13^, ^14^

Previous studies have identified that miR‐103 and miR‐107, belonging to the same family and only differ at one nucleotide residue near the 3′ end, are differentially expressed in CSF of AD patients and have evolutionary conserved binding sites for AD‐related gene, a disintegrin and metalloproteinase 10 (ADAM10).^15^, ^16^, ^17^ Moreover, miR‐103 is previously shown to promote neurite outgrowth and suppresses neuron apoptosis by targeting prostaglandin‐endoperoxide synthase 2 (PTGS2) in AD.^18^ Nonetheless, the predictive value of circulating miR‐103 and miR‐107 for AD susceptibility and progression is still unknown. Therefore, this study assessed the ability of plasma miR‐103 and miR‐107 to predict AD risk as well as their correlation with cognitive impairment in AD patients.

## 2 MATERIALS AND METHODS

### 2.1 Subjects

A hundred and twenty AD patients, 120 PD patients (served as disease control), and 120 healthy controls from Tongren Hospital, Shanghai Jiao Tong University School of Medicine, between January 2018 and December 2018 were recruited in this case‐control study. The diagnosis of AD was based on the criteria of National Institute of Neurological and Communication Disorders and Stroke/Alzheimer**'**s disease and Related Disorders Association (NINCDS‐ADRDA)^19^; the diagnosis of PD was made according to the criteria of Parkinson's Disease Society Brain Bank.^20^ And the healthy controls were neurologically healthy, as reflected by medical history, general examinations, laboratory examinations, and Mini‐Mental State Examination (MMSE). All enrolled subjects were older than 18 years, and both the PD patients and the healthy controls were matched with AD patients by age and gender, while the subjects were excluded from the study if they were complicated with other malignancies or hematological diseases, presenting with infection, pregnant, or breast‐feeding women. The present study was approved by the Ethics Committee of Tongren Hospital, Shanghai Jiao Tong University School of Medicine, and all enrolled subjects or their guardians provided written informed consents prior to the initiation of the study.

### 2.2 Data collection and assessment

When the eligibilities of subjects were confirmed and the written informed consents were collected, demographic data of all subjects were documented, including age, gender, as well as education duration. Meanwhile, the MMSE score was assessed and recorded as well. The MMSE is a 30‐question assessment of cognitive function evaluating attention and orientation, memory, registration, recall, calculation, language, and ability to draw a complex polygon, with a total possible score of 30 points.^21^ And the AD dementia severity based on the MMSE score was defined as follows: mild dementia: 21 ≤ MMSE score ≤ 26; moderate dementia: 15 ≤ MMSE score ≤ 20; and severe dementia: MMSE score < 15.^22^

### 2.3 Sample collection and determination

Peripheral blood samples were collected from all subjects after recruitment via vacuum blood collection tubes containing ethylenediaminetetraacetic acid (EDTA). Immediately, the plasma was separated by centrifugation at 800 *g* for 15 min (4°C) and stored at −80°C for the further detection. The expressions of miR‐103 and miR‐107 were determined by the reverse transcription‐quantitative polymerase chain reaction (RT‐qPCR).

### 2.4 RT‐qPCR

Total RNA was extracted from plasma using QIAamp RNA Blood Mini Kit (Qiagen) and reversely transcribed to cDNA by QuantiTect Rev. Transcription Kit (Qiagen). Following that, QuantiNova SYBR Green PCR Kit (Qiagen) was used to perform qPCR. The results were calculated by the 2^−ΔΔCt^ formula, and U6 was used as internal reference. Sequences of the primers applied in the RT‐qPCR were as follows: miR‐103, forward primer (5′ → 3′): ACACTCCAGCTGGGAGCTTCTTTACAGTGC, reverse primer (5′ → 3′): TGTCGTGGAGTCGGCAATTC; miR‐107, forward primer (5′ → 3′): ACACTCCAGCTGGGAGCAGCATTGTACAGG, reverse primer (5′ → 3′): TGTCGTGGAGTCGGCAATTC; U6, forward primer (5′ → 3′): CTCGCTTCGGCAGCACATATACTA, reverse primer (5′ → 3′): ACGAATTTGCGTGTCATCCTTGC.

### 2.5 Statistical analysis

Data were expressed as mean and standard deviation (SD), median and interquartile range (IQR), or count (percentage). Comparisons among groups were determined by the chi‐square test, one‐way analysis of variance (ANOVA), or Kruskal‐Wallis H test followed by the Benjamini‐Krieger‐Yekutieli test. Correlations between variables were determined by the Spearman's rank correlation test. The feasibilities of variables in discriminating different subjects were analyzed by plotting receiver operating characteristic (ROC) curve and calculating the area under the ROC curve (AUC) and the specificity and sensitivity at the median values of variables. All analyses were performed using SPSS 24.0 software (IBM) and GraphPad Prism 6.01 software (GraphPad Software Inc). *P* value < .05 was considered statistically significant.

## 3 RESULTS

### 3.1 Patients' characteristics

The mean age of healthy controls, PD patients, and AD patients was 71.2 ± 10.8 years, 70.5 ± 8.3 years, and 72.5 ± 7.7 years, respectively (Table 1). There was no difference in age (*P* = .217) or gender (*P* = .363) among healthy controls, PD patients, and AD patients, whereas, for education and MMSE score, healthy controls had the longest education duration, followed by PD patients and then AD patients (*P* = .004); MMSE score was the highest in healthy controls, the intermediate in PD patients, and the lowest in AD patients (*P* < .001).

**Table 1:** Characteristics of participants

Items	Healthy controls (N = 120)	PD patients (N = 120)	AD patients (N = 120)	*P* value
Age (y), mean ± SD	71.2 ± 10.8	70.5 ± 8.3	72.5 ± 7.7	.217
Gender, No. (%)				.363
Female	66 (55.0)	56 (46.7)	65 (54.2)	
Male	54 (45.0)	64 (53.3)	55 (45.8)	
Education duration (y), mean ± SD	6.7 ± 4.2	6.5 ± 3.5	5.3 ± 3.2	.004
MMSE score, mean ± SD	28.5 ± 0.6	26.8 ± 2.2	16.8 ± 3.0	<.001

Note

Comparison was determined by one‐way ANOVA or chi‐square test.

Abbreviations: AD, Alzheimer's disease; MMSE, Mini‐Mental State Examination; PD, Parkinson's disease; SD, standard deviation.

### 3.2 miR‐103 expression among AD patients, PD patients, and healthy controls

**Figure 1:**
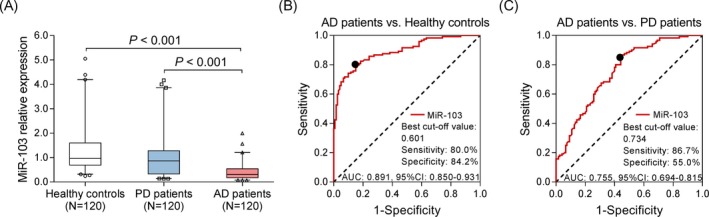
Expression of miR‐103. The expression of miR‐103 in healthy controls, PD patients, and AD patients (A). The value of miR‐103 in distinguishing AD patients and healthy controls (B). The value of miR‐103 in distinguishing AD patients and PD patients (C). Comparison among three groups was determined by Kruskal‐Wallis H rank sum test, and multiple comparisons were determined by Benjamini‐Krieger‐Yekutieli test. The feasibilities of variables in discriminating different subjects were analyzed by plotting ROC curve and calculating the AUC *P* < .05 was considered significant. AD, Alzheimer's disease; AUC, area under the curve; miR‐103, microRNA‐103; PD, Parkinson's disease; ROC, receiver operating characteristic

miR‐103 expression was lower in AD patients compared with PD patients (*P* < .001) and healthy controls (*P* < .001) (Figure 1A). The following ROC curve analyses displayed that miR‐103 presented great value in distinguishing AD patients from healthy controls with AUC of 0.891 (95% CI: 0.850‐0.931); the sensitivity and specificity were 80.0% and 84.2%, respectively, at the best cutoff point (miR‐103 = .601) (Figure 1B). miR‐103 also had relatively good value in differentiating AD patients from PD patients with AUC of 0.755 (95% CI: 0.694‐0.815); sensitively and specificity were 86.7% and 55.0%, respectively, at the best cutoff value (miR‐103 = .734) (Figure 1C).

### 3.3 Correlation of miR‐103 with MMSE score and dementia severity

**Figure 2:**
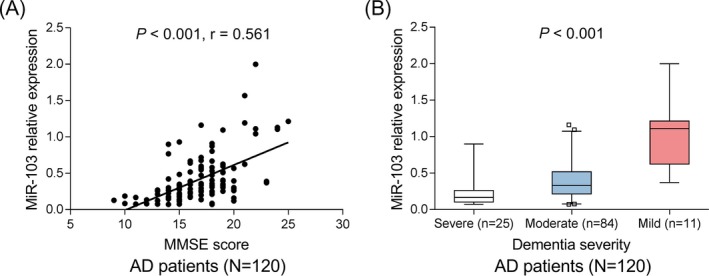
Correlation of miR‐103 with cognitive impairment in AD patients. Correlation of miR‐103 with MMSE score in AD patients (A). Correlation of miR‐103 with dementia severity in AD patients (B). Correlations were determined by Spearman's rank correlation test. *P* < .05 was considered significant. AD, Alzheimer's disease; miR‐103, microRNA‐103; MMSE, Mini‐Mental State Examination

miR‐103 expression was positively correlated with MMSE score in AD patients (*P* < .001, *r* = .561) (Figure 2A). As for dementia severity, miR‐103 expression was the highest in patients with mild dementia, followed by patients with moderate dementia, and the lowest in patients with severe dementia (*P* < .001), which indicated that miR‐103 was negatively correlated with dementia severity in AD patients (Figure 2B).

### 3.4 miR‐107 expression among AD patients, PD patients, and healthy controls

**Figure 3:**
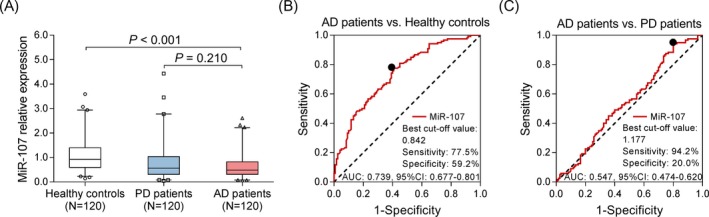
Expression of miR‐107. The expression of miR‐107 in healthy controls, PD patients, and AD patients (A). The value of miR‐107 in distinguishing AD patients and healthy controls (B). The value of miR‐107 in distinguishing AD patients and PD patients (C). Comparison among three groups was determined by Kruskal‐Wallis H rank sum test, and multiple comparisons were determined by Benjamini‐Krieger‐Yekutieli test. The feasibilities of variables in discriminating different subjects were analyzed by plotting ROC curve and calculating the AUC *P* < .05 was considered significant. AD, Alzheimer's disease; AUC, area under the curve; miR‐107, microRNA‐107; PD, Parkinson's disease; ROC, receiver operating characteristic

miR‐107 expression was lower in AD patients compared with healthy controls (*P* < .001) but similar between AD patients and PD patients (*P* = .210) (Figure 3A). The following ROC curve analyses displayed that miR‐107 presented good value in distinguishing AD patients from healthy controls with AUC of 0.739 (95% CI: 0.677‐0.801); the sensitivity and specificity were 77.5% and 59.2%, respectively, at the best cutoff point (miR‐107 = 0.842) (Figure 3B). However, the value of miR‐107 for differentiating AD patients from PD patients was poor with AUC being 0.547 (95% CI: 0.474‐0.620) (Figure 3C). These indicated that miR‐107 might not be suitable to be a specific biomarker for AD risk.

### 3.5 Correlation of miR‐107 with MMSE score and dementia severity

**Figure 4:**
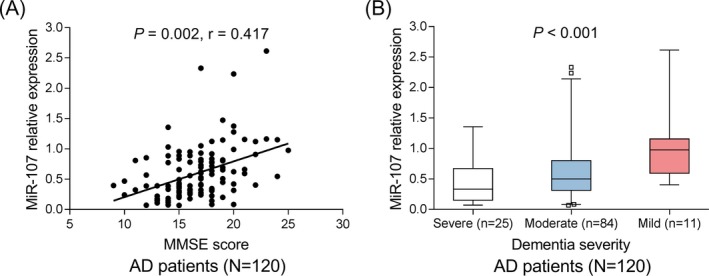
Correlation of miR‐107 with cognitive impairment in AD patients. Correlation of miR‐107 with MMSE score in AD patients (A). Correlation of miR‐107 with dementia severity in AD patients (B). Correlations were determined by Spearman's rank correlation test. *P* < .05 was considered significant. AD, Alzheimer's disease; miR‐107, microRNA‐107; MMSE, Mini‐Mental State Examination

miR‐107 expression was positively associated with MMSE score in AD patients (*P* = .002, *r* = .417) (Figure 4A). Regarding dementia severity, patients with mild dementia exhibited the highest miR‐107 expression, followed by patients with moderate dementia and then patients with severe dementia (*P* < .001), which implied that miR‐107 was negatively correlated with dementia severity in AD patients (Figure 4B).

### 3.6 Correlation between miR‐103 and miR‐107

**Figure 5:**
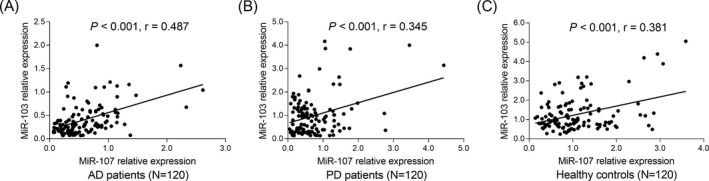
Positive correlation between miR‐103 and miR‐107. The correlation between miR‐103 and miR‐107 in AD patients (A), PD patients (B), and healthy controls (C). Correlations were determined by Spearman's rank correlation test. *P* < .05 was considered significant. AD, Alzheimer's disease; miR‐103, microRNA‐103; miR‐107, microRNA 107; PD, Parkinson's disease

Positive correlation was observed between miR‐103 and miR‐107 in AD patients (*P* < .001, *r* = .487) (Figure 5A), PD patients (*P* < .001, *r* = 0.345) (Figure 5B), and healthy controls (*P* < .001, *r* = .381) (Figure 5C).

## 4 DISCUSSION

miR‐103 was more suitable than miR‐107 to serve as a biomarker for decreased AD susceptibility, and both miR‐103 and miR‐107 were negatively correlated with cognitive impairment in AD patients.

As a typical neurodegenerative disease, AD is a progressive disorder that attributes to multiple risk factors including genetic, environmental, and epigenetic mechanisms.^5^ Thus, miRNAs that function as important epigenetic regulators have attracted much attention as biomarkers for AD risk with several miRNAs being identified in the nervous system to influence neurogenesis, dendritic outgrowth, dendritic spine formation, etc, probably via regulating the mRNAs encoding the toxic proteins and mediating neural cell proliferation and apoptosis.^9^ Furthermore, the contributions of miRNAs to AD development and progression have also been demonstrated to largely rely on their ability to alter the expression of toxic protein‐coding genes. For instance, miR‐140‐5p expression is enhanced in the AD postmortem brain hippocampus, and it downregulates ADAM10 that poses neuroprotective effect in early AD.^23^, ^24^ The expression of miR‐29 is decreased in AD patients who have high levels of human β‐secretase, and employment of miR‐29 suppresses human β‐secretase‐induced Aβ peptide.^25^ In addition, certain miRNAs regulate genes involved in neuroinflammation and chronic neurodegeneration.^12^, ^26^ For instance, miR‐424 level is inversely correlated with neurotrophic factor, which targets neuroinflammation to protect neuroprotection in a mouse model.^27^, ^28^ These studies establish the importance of miRNAs in pathogenesis of AD and illuminate the potential of miRNAs as AD biomarkers.

As for miR‐103 and miR‐107, a study conducted in cellular model of AD exhibited that miR‐103 promotes neurite outgrowth and suppresses cell apoptosis by targeting PTGS2.^18^ And miR‐107 upregulation is shown to facilitate cell survival, reduce lactate dehydrogenase leakage, and inhibit apoptosis and Aβ production in AD.^29^ Besides, expression of miR‐107 is decreased even in early stage of AD, and its downregulation accelerates disease progression via mediating β‐site amyloid precursor protein‐cleaving enzyme 1.^30^ The above evidence suggests that miR‐103 and miR‐107 both suppress pathological progression in AD; however, their potential to predict AD risk is still unknown. In this study, we observed that miR‐103 was able to differentiate AD patients from PD patients and healthy controls; miR‐107 was able to differentiate AD patients from healthy controls but not AD patients from PD patients. Here are several possible reasons: (a) Suppression of miR‐103 may inhibit neurite outgrowth and promote neuron apoptosis that increased AD risk, and miR‐103 in CSF is also shown to target AD‐related genes such as beta‐secretase 1 (BACE1) and RE1 silencing transcription factor; therefore, decreased miR‐103 may raise susceptibility to AD.^15^ (b) miR‐107 regulates post‐transcription of BACE1 by targeting the 3′‐UTR of BACE1 mRNA; thereby, miR‐107 downregulation may lead to increased BACE1 and the subsequent cleavage of Aβ precursor protein that generates neurotoxic Aβ peptide, which contributes to AD risk.^30^ However, miR‐107 failed to distinguish AD patients from PD patients, which reduced its value as a biomarker for AD.

Cognitive impairment is a pronounced symptom of AD, and it gets worse as the disease progresses.^31^ MMSE is a neuropsychological examination consisting of a series of questions and cognitive tests that are commonly used to assess the cognitive impairment and degree of dementia.^32^ In our study, we evaluated the correlations of miR‐103 and miR‐107 with MMSE score in AD patients and observed that both miR‐103 and miR‐107 were positively correlated with MMSE score and negatively associated with dementia severity in AD patients, which indicated that miR‐103 and miR‐107 might attenuate the disease progression of AD. This can be explained by that miR‐103 and miR‐107 suppress the translation of toxic proteins responsible for AD pathogenesis via regulating the gene transcription, thus attenuating the disease progression of AD and thereby reduce the degree of cognitive impairment in AD patients. Nevertheless, the correlation coefficient of miR‐107 with MMSE score was lower than that of miR‐103 with MMSE score. And this further supported that miR‐103 might be a better choice than miR‐107 as a biomarker for AD progression. In addition, miR‐103 was positively correlated with miR‐107 in AD patients, which was in line with the previous evidence.^30^

A limitation of this study was the relatively small sample size, which was a challenge that most of the clinical studies would face. Besides, although the potential of miR‐103 as AD biomarker was revealed in this study, there was still a huge gap between our findings and clinical application of miR‐103 in assisting diagnosis of AD, which needed to be solved by additional mechanism investigations and large‐scale clinical studies.

In conclusion, circulating miR‐103 is a better choice than miR‐107 to serve as a potential biomarker for disease risk and disease progression for AD.

## CONFLICT OF INTEREST

The authors declare that there are no conflicts of interest.
